# Updates in Sertoli Cell-Mediated Signaling During Spermatogenesis and Advances in Restoring Sertoli Cell Function

**DOI:** 10.3389/fendo.2022.897196

**Published:** 2022-05-04

**Authors:** Victor A. Ruthig, Dolores J. Lamb

**Affiliations:** ^1^Department of Urology, Weill Cornell Medicine, New York, NY, United States; ^2^Sexual Medicine Lab, Weill Cornell Medicine, New York, NY, United States; ^3^Center for Reproductive Genomics, Weill Cornell Medicine, New York, NY, United States

**Keywords:** sertoli cell (SC) niche, transitional zone (TZ), Sertoli cell ablation, Sertoli cell transplantation, Spermatogenesis, FSH signaling, AR signaling, Exosome extracellular vesicle (EV)

## Abstract

Since their initial description by Enrico Sertoli in 1865, Sertoli cells have continued to enchant testis biologists. Testis size and germ cell carrying capacity are intimately tied to Sertoli cell number and function. One critical Sertoli cell function is signaling from Sertoli cells to germ cells as part of regulation of the spermatogenic cycle. Sertoli cell signals can be endocrine or paracrine in nature. Here we review recent advances in understanding the interplay of Sertoli cell endocrine and paracrine signals that regulate germ cell state. Although these findings have long-term implications for treating male infertility, recent breakthroughs in Sertoli cell transplantation have more immediate implications. We summarize the surge of advances in Sertoli cell ablation and transplantation, both of which are wedded to a growing understanding of the unique Sertoli cell niche in the transitional zone of the testis.

## Introduction

**Figure 1:**
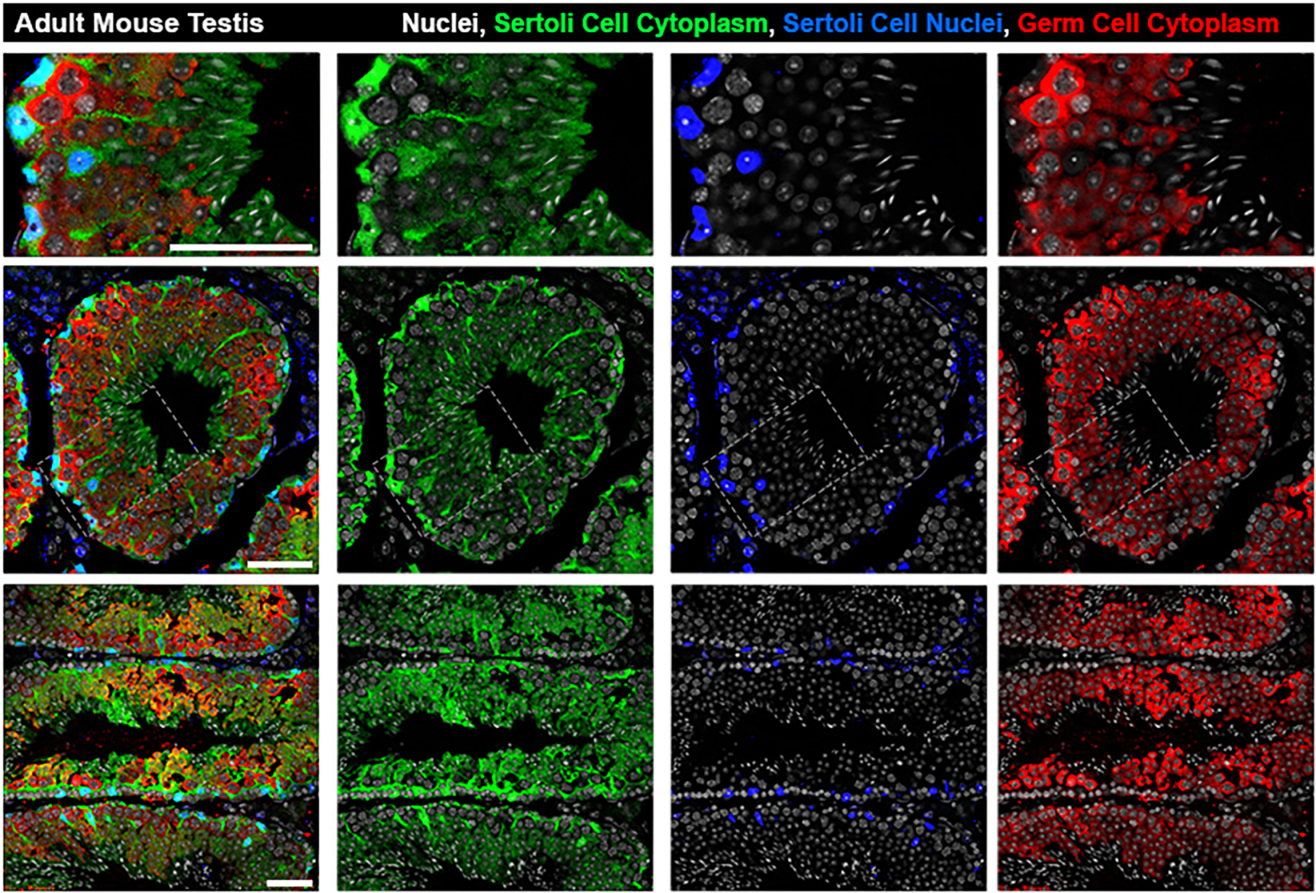
Architecture of Sertoli cells in the adult mouse seminiferous tubule. The bodies of Sertoli cell cytoplasm (green) can be seen engulfing germ cells (red) from basal lamina to lumen while Sertoli cell nuclei (blue) are located basally. Top row: zoomed inset from grey boxed region in Middle Row: seminiferous tubule cross section at stage V-VI. Bottom Row: Longitudinal sections showing multiple stages. All scale bars are 50µm.

Although germ cells are the stars of spermatogenesis, Sertoli cells are the sustaining lead, without which, spermatogenesis would cease to occur. Sertoli cells provide the supportive framework within which germ cells will safely undergo rounds of mitosis and meiosis (**Figure 1**). This structure which includes tight junctions between adjacent Sertoli cells, divides the seminiferous epithelium into the basal and adluminal compartments, serving a protective role as the testicular region within the seminiferous tubules that is immuno-privileged (1–5). Sertoli cells act as the mediator between germ cells and endocrine signaling, from controlling spermatogenesis by hormones (follicle stimulating hormone [FSH] and testosterone [T]), originating from outside of the seminiferous tubule (6–8). Sertoli cells also have direct impacts on germ cell development through paracrine signaling (9–11). These roles are all key elements required to orchestrate the symphonic cyclicity of steady-state spermatogenesis within the adult testis. When aberrations in Sertoli cell function occur, this intricate exchange breaks down and spermatogenic failure may occur, ultimately challenging the fertility of the male. Recent research into the niche population of Sertoli cells at the transition zone between the rete testis and seminiferous tubules, as well as studies of Sertoli cell transplantation, are bringing new insights to the field. Both branches of investigation offer the promise of a deeper understanding into how Sertoli cells come to reside properly in the testis, and methods for getting functional Sertoli cells in to replace Sertoli cells that are deficient.

**Figure 2:**
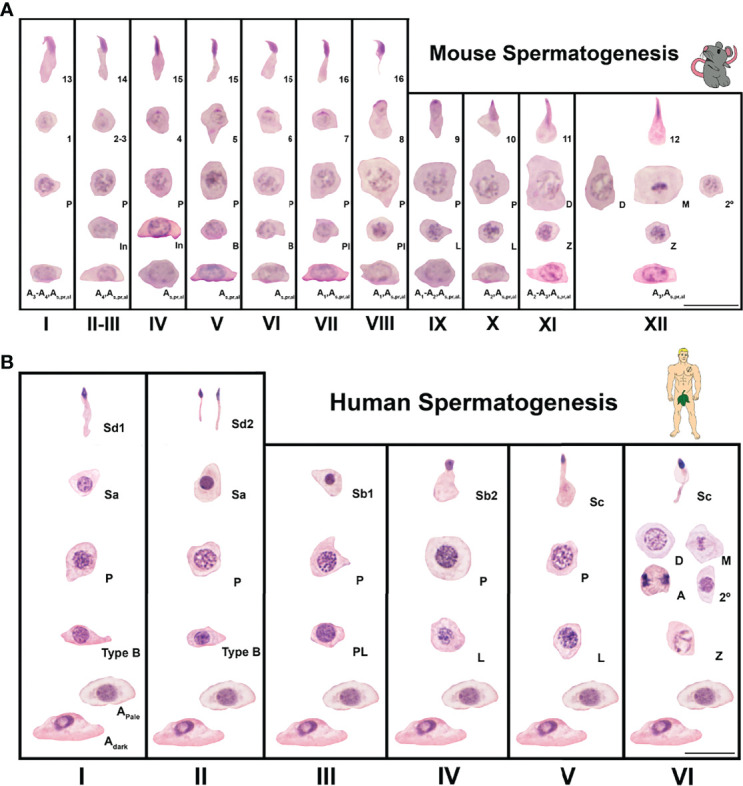
Seminiferous epithelial stages of mouse and human spermatogenesis as classic spermatogenesis cycle staging charts using germ cell associations and morphology. Spermatogenesis is the process of sperm development and involves phases of mitosis, meiosis, and spermiogenesis (morphological cell changes). **(A)** Spermatogenesis in mice is a cycle that takes ~8.6 days (12–14). The time necessary for a germ cell to go from type A spermatogonia to spermatozoa (the complete process or duration of spermatogenesis) is about 35 days (12, 13, 15). In mice, spermatogenesis is divided into 12 stages (I-XII) and 16 spermatid developmental steps. A, In, and B are type A, intermediate, and type B spermatogonia, respectively. Pl, L, Z, P, D, M, and 2º are preleptotene, leptotene, zygotene, pachytene, diplotene, meiotic, and secondary spermatocytes, respectively. Steps of spermatid development are numbered 1-16. Sections were stained with Periodic Acid Schiff’s regent-hematoxylin (PAS-H), which is a conventional staining for staging of mouse testis sections. Scale is 20μm. **(B)**. Spermatogenesis in men is a 16 day cycle with a complete duration that was classically determined to be 64 days but modern methods show to be closer to 74 days (16–21). In humans, spermatogenesis is divided into 6 stages (I-VI) and 6 spermatid developmental steps. Adark, Apale and B are type A dark, type A pale and type B spermatogonia, respectively. Pl, L, Z, P, D, M, A and 2º are preleptotene, leptotene, zygotene, pachytene, diplotene, meiotic metaphase, meiotic anaphase and secondary spermatocytes, respectively. Steps of spermatid development are labeled Sa, Sb1, Sb2, Sc, Sd1 and Sd2. Sections were stained with Periodic Acid Schiff’s regent-hematoxylin (PAS-H), which is a conventional staining for human testis histology assessment. Scale is 20μm.

Aside from the germ cell based histological staging of spermatogenesis defined by consistent cell associations present in cross-sections of the seminiferous tubule (**Figure 2**), generally the stages of the cycle can also be defined by unique metabolic and molecular Sertoli cell identities (22–24). Specifically in regards to the androgen signaling pathway, Sertoli cells display stage specific temporal peaks of AR expression in rodents (stages VI-VIII) (25–27) (**Figure 2A**), and humans (stage III) (28) (**Figure 2B**). For germ cells, as one progresses concentrically towards the seminiferous tubule lumen, this AR peak period coincides with: undifferentiated type A spermatogonia meiotic entry, elongating spermatid adhesion, and spermiation (29–32).

## Endocrine and Paracrine Signals

Larose et al. 2020 (33) took a more granular look at the direct impact of AR presence in Sertoli cells on germ cell meiotic progression. Using SCARKO mutant mice (Sertoli cell androgen receptor knockout) they defined a Sertoli cell-AR androgen independent period of germ cell development from meiotic initiation to early prophase. Germ cells in these mice that did not undergo apoptosis (and many germ cells did) progressed up to what, histologically, appeared to be relatively normal pachytene spermatocytes. But upon deeper investigation using scRNA-seq, the most advanced germ cells were transcriptionally defined and resembled leptotene or zygotene spermatocytes (33). This discrepancy between transcriptomic and histological cell-identity was also reported in *Pdrm9* mutant germ cells (34). This finding calls into question the many definitive studies using models of androgen deficiency or receptor deletion causing a defined maturation arrest that predates the use of scRNA-seq technology and relied solely on classical histological assessment. Revisiting these classic maturation arrest studies with modern bioinformatics tools has the potential to elucidate other molecular details similar to those reported by 33.

Transcriptomic analysis on SCARKO mutant mice also identified a set of genes (including: *Fabp9*, *Gstm5*, *Ybx3*, *Meig1*, *Spink2*, *Rsph1*, *Aldh1a1*, *Igfbps*, *Piwil1*, *Mael*) regulated by AR signaling in Sertoli cells. Collectively this gene set seems to license spermatocytes for the first meiotic division, as well as for spermiogenic competency (33). Another gene, *Rhox5*, initially transcribed in Sertoli cells, is an androgen-inducible transcription factor (35–39). RHOX5 regulates Sertoli cell gene expression controlling cell surface and protein secretion in relation to germ cells (7, 40–43). *Rhox5* has two promoters, distal and proximal. Previously, these promoters were understood to drive different tissue-specific expression, with the exception that both promoters are active in adult Sertoli cells Bhardwaj et al. 2022 defined a postnatal temporality to *Rhox5* promoter activity (44). The proximal promoter is activated shortly after birth, while the distal promoter is dormant until late in the postnatal period also identified novel androgen-responsiveness for the *Rhox5* distal promoter. The group then established that the proximal promoter can act as an enhancer for the distal promoter and further, that RHOX5 up-regulates its own transcription *via* the distal promoter (44).

*Rhox5* expression in Sertoli cells is dependent on FSH signaling (36). Unlike *Ar*, in adult mouse Sertoli cells *Fshr* has a consistent expression level throughout the stages of spermatogenesis (23) and knockout experiments have shown there is a degree of added redundancy in the FSH pathway when working synergistically with the AR pathway (45, 46). Reported activity of both proximal and distal *Rhox5* promoters into adulthood specifically in Sertoli cells at Stages II-V (outside AR peak) and VI-VIII (within AR peak) (44). Potentially, *Rhox5* is yet another recipient of synergistic T and FSH action. This would add another layer to the evolutionary pressure postulated by 44. According to the authors, this pressure drove retention of the *Rhox5* distal and proximal promoters. This evolutionary pressure was probably directed at the initial temporally-staggered promoter expression of *Rhox5* postnatally. During the first wave of spermatogenesis, *Ar* and *Fshr* are known to have dynamic expression patterns in mouse Sertoli cells (24, 44).

T and FSH synergism is not limited to Sertoli cell transcription factors. A newer player in the realm of intercellular signaling is the extracellular vesicle, which can hold and transport an array of different molecules including: growth factors, cytokines, mRNAs, bioactive lipids, and microRNAs (47–49). A recent report by Mancuso et al 2015 utilized a porcine Sertoli cell culture system to define the extracellular vesicle components with FSH-alone and synergistic T+FSH stimulation (50). Proteomic analysis showed FSH-alone increased proteins generally linked to modulating the hypothalamic-pituitary axis regulating testosterone biosynthesis, the blood-testis-barrier, and spermiation (INHA, INHB, PLKA, HPT, SERA and AT1A1). While stimulation (50) with T+FSH increased proteins generally linked to blood-testis-barrier adherens junctions, and gating endocrine and paracrine regulation of spermatogenesis (INHA, INHB, TPA, EGFL8, EF1G and SERA). These extracellular vesicles also contained transcripts (*Amh*, *Inhb*, *Abp*, *Fshr*), which the authors postulate could function in loading germ cells, and other testicular cells, with mRNA that will later be translated (50).

Extracellular vesicles are generally accepted to belong to 3 categories: exosomes, microvesicles, and apoptotic bodies (51, 52). Exosomes, were recently the focus of exciting findings in the field. Aside from transporting mRNA, extracellular vesicles, specifically exosomes, can also transport microRNA (53). Paracrine signaling from Sertoli to germ cells by exosomes containing microRNA would putatively be to silence genes. Indeed, a recent report by Li et al. 2021, revealed that Sertoli exosomes contain the microRNA miR-486-5p (54). The authors used a culture system of adult Sertoli cells and P6 germ cells enriched for spermatogonial stem cells. Using this system demonstrated that Sertoli cell exosomes with miR-486-5p down-regulated spermatogonial stem cell expression of *Pten* by targeting of the Pten-3’UTR by miR-486-5p. The authors further identified that both *Stra8*. and *Sycp3* were indirectly up-regulated in spermatogonial stem cells by the decrease in repressive PTEN. Ultimately this exosome exchange would seem to be part of the differentiation signal from Sertoli cells to spermatogonia (54).

The observations of Li et al. 2021 about Sertoli cell miR-486-5p containing exosomes adds to the evolving school of thought on how undifferentiated spermatogonia enter meiosis (54). Spermatogonial differentiation and meiotic entry is established to be highly dependent on retinoic acid (RA) signaling (55, 56). The commonly proposed paracrine source of germ cell stimulating RA is Sertoli cells and spermatocytes (32, 57–60). Much like AR, RA levels in the seminiferous epithelium are also cyclic and peak at stage VIII, the same stage at which undifferentiated spermatogonia commit to meiosis (61). Timing for meiotic entry is critically important, and inherent in understanding the control of this timing is the need to define how spermatogonia control RA-responsiveness. In the fetal testis CYP26B1, which catabolizes RA, is a key regulator in blocking fetal male germ cell meiotic entry (62–65). Using the first wave of spermatogenesis as a synchronized model of spermatogenesis, Velte et al. 2019 (66) showed that CYP26 also blocks meiotic entry at postnatal day 6 (P6) in undifferentiated spermatogonia that are poised to respond to RA. Spermatogonial poising for RA responsiveness is generally thought to be accomplished through RARG (RA receptor gamma) expression (66). Indeed, this model was eloquently validated by in Suzuki et al. (67), who defined two sub-populations of undifferentiated spermatogonia in the adult mouse testis. Early-undifferentiated spermatogonia did not express RARG, while late-undifferentiated spermatogonia did express RARG (67). However deeper analysis in a follow-up study further sub-divided late-undifferentiated spermatogonia into a group expressing *Dppa3* (*Dppa3*+) and RARG that quickly transition to a differentiating spermatogonia (KIT+) state upon RA stimulation. While the other group of late-undifferentiated spermatogonia express RARG but not *Dppa3* (*Dppa3*-) and have delayed differentiation (68). Whether or not *Dppa3* transcript presence is the product of exosome-mediated microRNA silencing is still an open question.

## Sertoli Cell Transplantation and Transitional Zone Sertoli Cell Niche

Clinically, men can suffer from an array of Sertoli cell-origin infertility. In some cases the ligand is the issue: gonadotropin-deficient men, mutations (69) and androgen dysregulation (70). In other cases the receptor is the issue, such as complete or partial androgen insensitivity syndromes resulting from polymorphisms or deletions of the androgen receptor (71, 72). Extracellular vesicles may offer the possibility of a cell-free treatment for some forms of infertility due to specific types of Sertoli cell deficiencies. Theoretically extracellular vesicles could be injected clinically through the rete testis using the ultrasound-guided injection technique (73–76). Although these types of therapeutics are still years away, extracellular vesicles could become clinically relevant sooner due to their diagnostic potential. Two recent studies demonstrated the value of seminal exosome analysis as markers of Sertoli cell damage by varicocele (77), and predictive of testicular sperm presence in NOA men (78).

Another exciting technology that has seen a surge of progress lately is Sertoli cell transplantation. Ralph Brinster pioneered germ cell transplantation over a quarter century ago, his technique was later applied to transplant the somatic cells of the seminiferous epithelium, Sertoli cells (79). Some of the earliest reporting of Sertoli cell transplantation as a method for repairing the spermatogonial stem cell niche goes back to the early 2000’s (80, 81). A challenge to restoring Sertoli cell function through transplantation of functional Sertoli cells is what to do about clearing out the dysfunctional Sertoli cells from the seminiferous epithelium to make space. Previously transgenic lines and cadmium has been used for Sertoli cell ablation (81–84). Although effective, from a clinical perspective these methods are not feasible and pose adverse risks, respectively.

Yokonishi et al. 2020 (85) recently identified a safe alternative to cadmium, benzalkonium chloride (BC), which is an FDA-approved non-toxic agent present in over-the-counter eye drops and hair conditioner (86). The authors show that admission of 0.02% benzalkonium chloride through the mouse rete testis is sufficient to ablate Sertoli cells. Further this group defines the temporal windows for host Sertoli cell ablation, donor Sertoli cell transplantation, and donor germ cell transplantation. The window for host germ cell survival is also detailed, the method is tested with cryopreserved testicular cells, and a culture version of the method demonstrates benzalkonium chloride utility in large mammals (dog) (85). In a follow-up study the same group shower that fetal mouse gonadal cells transplanted into an ablated adult mouse testis are competent to colonize, mature, and support host germ cell spermatogenesis (87). An added level of temporality in transplanted donor Sertoli cell colonization after ablation, was recently defined in another robust ablation study. Using a transgenic system of Sertoli cell ablation, Imura-Kishi et al. 2021 showed that donor Sertoli cells first colonize the transitional zone where they resume repression of spermatogenesis. After reaching an equilibrium in the transitional zone Sertoli cells then proliferate further, repopulating the host seminiferous epithelium where the donor Sertoli cells will support host spermatogenesis (88).

The transitional zone of the testis goes by many names (Sertoli valve, transitional region, tubulis rectus, intermediate region, terminal segment) expertly reviewed in (89). Foundation papers first describing this area between the rete testis and spermatogenic seminiferous epithelium date back to the 60’s (90–97). Sertoli cells in the transitional zone are morphologically distinct having long string-like cell bodies that extend distally into the rete testis, structurally giving the zone a valve appearance histologically (98). At least a sub-population of these transitional zone Sertoli cells has been documented by multiple labs to be proliferatively competent (99–103). Specifically, because some transitional zone Sertoli cells do not express the maturation markers p27, GATA4 and AR (101). AR is not just a marker for Sertoli cell maturation and proliferative cessation (104, 105). Loss of AR has been shown to inhibit Sertoli cell maturation (106). In men and rodents, germ cells that reside in this region are exclusively spermatogonia that seem to be predominantly undifferentiating spermatogonia (88, 92, 99, 107). Collectively the transitional zone represents a unique Sertoli-germ cell niche within the testis.

During their ablation experiments, Imura-Kishi et al. 2021, identified transitional zone Sertoli cell *Cyp26a1* expression that is at least partially responsible for blocking RA signaling to the spermatogonia in the transitional zone. Due to the proximity to the rete testis, the authors also showed retrograde rete derived FGF signaling may also competitively inhibit RA signal in the transitional zone (88). A separate recent report defined two sub-populations of transitional zone Sertoli cells that were KRT8+,DMRT1- or KRT8+,DMRT1+ (108). DMRT1 is essential in differentiation of Sertoli cells into a non-proliferative state (109). These studies elucidated the molecular uniqueness of the transitional zone niche, but there is still much we do not understand about cell identity and function in the transitional zone. Given the recent reports on exosomes, one cannot help but wonder if there is also a unique population of transitional zone Sertoli cell extracellular vesicles that are part of maintaining this niche.

## Discussion

Ablation and transplantation are done *via* injection through the rete testis (110). Even when done by the most skilled pair of hands, this represents a traumatic event to the surrounding tissue. The plasticity of the Sertoli cell population in the transitional zone and the robustness of this epithelium is a fortunate coincidence for this method, but also represents an intriguing source for discoveries in reversing Sertoli cell dysfunction and repopulating a Sertoli cell deficient testis. Sertoli cells in human testes partially resume proliferation after gonadotropin suppression with coincident reduction of AR (111). Continued research into maintenance and control of proliferative transitional zone Sertoli cells in conjunction with Sertoli cell transplantation has the potential to unlock new therapeutics for treatment of Sertoli cell based male infertility, and reversing the reproductive harm done by gonadotoxic cancer treatment.

## Author Contributions

VAR generated the direction for the manuscript, and produced the figures. DJL supervised the creative process providing expert feedback and insight. VAR and DJL wrote the manuscript and reviewed the manuscript. All authors contributed to the article and approved the submitted version.

## Conflict of Interest

DJL serves on the Ro advisory board, and as a consultant, and has equity; and for Fellow has equity; and serves as Secretary-Treasurer for the American Board of Bioanalysts with honorarium.

The remaining author declares that the research was conducted in the absence of any commercial or financial relationships that could be construed as a potential conflict of interest.

## Publisher’s Note

All claims expressed in this article are solely those of the authors and do not necessarily represent those of their affiliated organizations, or those of the publisher, the editors and the reviewers. Any product that may be evaluated in this article, or claim that may be made by its manufacturer, is not guaranteed or endorsed by the publisher.
